# The middle-term outcome of carotid endarterectomy and stenting for treatment of ischemic stroke in Chinese patients

**DOI:** 10.1038/s41598-018-23061-7

**Published:** 2018-03-16

**Authors:** Lin Yang, Jianlin Liu, Guangyu Qi, Yanzi Li, Yamin Liu

**Affiliations:** 1 Department of Vascular Surgery, First Affiliated Hospital of Xi’an Jiaotong University, Xi’an, China; 2 Department of Surgery Operation, First Affiliated Hospital of Xi’an Jiaotong University, Xi’an, China

## Abstract

This study aims to investigate the complication and middle-term outcome of carotid endarterectomy (CEA) and carotid artery stenting (CAS) in Chinese patients, which was a retrospective case-control study and perioperative complications and 2-year end points were analyzed. Follow-up was done by a certified doctor, and restenosis was detected by ultrasound. Operation success rate were 100% in two groups. CAS showed the higher incidence rate of all stroke/TIA at 30days post-procedure (7.89% VS 1.85%, *P* = 0.038), odds ratio (OR) with 95% confidence interval, 4.54 (1.09–18.97), but there was no difference in the incidence rate of stroke subgroups, mortality and myocardial infarction between two groups. The higher incidence of hypertension with CEA (14.42% VS 5.26%, *P* = 0.012), OR: 2.90 (1.26–6.65) and hypotension with CAS (14.91% VS 1.85%, *P* = 0.001), OR: 0.11 (0.03–0.42). No difference in all stroke, ipsilateral stroke and mortality between two groups at 24 months post-procedures, however, the total incidence rate of stroke/death was higher in CAS (12.84% VS 4.72%, *P* = 0.036), OR: 2.98 (1.08,8.23). Higher restenosis rate of CAS was examined (13.76% VS 5.66%, *P* = 0.045), OR: 2.66 (1.02, 6.74). CAS and CEA showed a similar middle-term outcome, but CAS showed a higher incidence rate of stroke and restenosis after operation.

## Introduction

Ischemic stroke is characterized by high mortality and morbidity, and according to epidemiology data, carotid stenosis is an independent high-risk factor for ischemic stroke, with extracranial cerebrovascular stenosis causing 8% to 29% of all ischemic strokes^1–4^. Carotid endarterectomy (CEA) was initially proposed as the gold standard for the treatment of symptomatic carotid stenosis (≥70%) since it was established by Easctcott^5^. Carotid artery stenting (CAS) is a less invasive and effective treatment alternative for carotid stenosis, it eliminates the potential risk of general anesthesia and the local surgical complications of CEA, such as neck hematoma and cranial nerve injury^6^.

In recent decades, with developments in technology and experience, several large-scale randomized clinical trials (RCTs) and clinical comparative studies have reported varying outcomes^3–7^. But the meta-analyses in both symptomatic and asymptomatic patients have demonstrated superiority of CEA over CAS. These studies showed that CEA has a higher incidence of myocardial infarction (MI), while CAS is associated with a higher incidence of stroke^8^. However, these studies differed in terms of patient selection, experience of the surgeons, clinical parameters, etc.^9^. Therefore, the true effects of CEA and CAS should be further evaluated, and the debate must continue. Moreover, there have been few reports on CAS and CEA outcomes in Chinese patients. Specifically, data are lacking regarding the results of the two procedures for the same experienced team. In our study, the CEA and CAS procedures were performed by the same experienced vascular team, and we compared the complication rates of both operations and the mid-term endpoint results of both methods.

## Results

### Baseline data

A total of 248 patients were retrospective assessed, as intended. Of these patients, 108 underwent CEA and 114 underwent CAS. There were no significant differences in demographics or baseline data between the two groups (Table 1). There were 67 patients with stroke and 41 patients with intermittent TIA in the CEA group, and 77 patients with stroke and 37 patients with intermittent TIA in CAS. The rates of high-risk factors were as follows: i) the incidence of smoking: 63.89% in CEA and 71.05% in CAS; ii) the incidence of hypertension: 60.19% in CEA and 65.79% in CAS; iii) type 2-diabetes: 28.70% in CEA and 26.32% in CAS; iv) CAD: 14.81% in CEA and 15.79% in CAS; v) PAD: 12.04% in CEA and 14.04% in CAS; vi) hyperlipidemia: 27.78% in CEA and 29.82% in CAS; and vii) hyperhomocysteinemia: 77.78% in CEA and 74.56% in CAS.

**Table 1:** Demographics and baseline data in two groups.

	CEA (n = 108)	CAS (n = 114)	*P* value
Gender(F/M)	24/84	30/84	0.48
Age(y)	63.31 ± 8.54	65.01 ± 7.59	0.12
Weight(Kg)	67.25 ± 8.69	66.65 ± 8.89	0.51
Stroke group			0.39
Stroke	67	77	
TIA	41	37	
Smoking	69	81	0.25
Hypertension	66	75	0.39
2-diabetes	31	30	0.69
CAD	16	18	0.84
PAD	13	16	0.66
Hyperlipidemia	30	34	0.74
H-HCY	84	85	0.57

CEA:carotid endarterectomy; CAS:carotid artery stenting; TIA: transient ischemic attack; CAD: Coronary atherosclerotic disease; PAD, peripheral arterial disease; H-HCY:Hyperhomocysteinemia.

### Results of Operation

The technical success rate was 100%. There were 87 patients who underwent primary closure CEA (80.56%) and 21 who underwent patch closure (19.44%). Carotid shunts were used in 64 patients (59.26%), with the blocking time of carotid being 18.11 ± 3.55 min and the operation time being 56.06 ± 10.46 min.

All of the patients underwent balloon angioplasty and carotid stenting placement; the technical success rate was 100%. Every patient underwent pre-dilation (100%), and 23 patients (20.18%) underwent post-dilation after stenting. An open-cell stent was used in 99 patients (86.84%) and close-cell stents were used in 15 patients (13.16%). Sixty-six patients (57.89%) received a straight stent, while 48 (42.11%) received a tapered stent.

### Complications of procedures

The complications after the procedures are listed in Table 2. CHS occurred in both groups, and there was no significant difference between CEA and CAS procedures in this regard (11.11% VS 13.16%, *P* > 0.05). Most patients showed a slight headache and recovered completely without medical treatment, only a small number of patients received medical treatment and recovered completely. However, the incidence of hypertension was higher in the CEA group (14.42% VS 5.26%, *P* = 0.012), and the odds ratio (OR) was 2.90 with 95% confidence interval (CI, 1,26–6.65); while that of hypotension was lower (1.85% VS 14.91%, *P* < 0.01) after revascularization, the OR was 0.11 with 95% CI (0.03–0.42). The incidence of wound- and puncture-related complications were similar for the CEA and CAS groups (*P* > 0.05). No deep vein thrombosis or infection was detected after the procedures. Although the incidence of cranial nerve palsy and ICA occlusion was not significantly different between the two groups, one patient (0.98%) in the CEA group experienced temporary hoarseness, which recovered 3 months after the operation. One patient (0.88%) in the CAS group experienced occlusion of ICA at 4 days after the procedures, and this patient underwent emergency thrombolysis (Urokinase) and timely thrombectomy, however, the patient still died at 10 days after the operation.

**Table 2:** The complications after procedures.

N (%)	CEA (n = 108)	CAS (n = 114)	*P* value*	OR (95% CI)
CHS	12 (11.11)	16 (13.16)	0.64	N/A
Hypotension	2 (1.85)	17 (14.91)	<0.01	0.11 (0.03–0.42)
Hypertension	15 (14.42)	6 (5.26)	0.012	2.90 (1.26–6.65)
Hematoma	5 (4.63)	11 (9.65)	0.15	N/A
Cranial nerve palsy	1 (0.93)	0 (0)	0.98	N/A
ICA occlusion	0 (0)	1 (0.88)	0.98	N/A
DVT/Infection	0	0	Null	N/A

CEA: Carotid endarterectomy; CAS: Carotid artery stenting; CHS Cerebral hyperperfusion syndrome; ICA: Internal Carotid Artery; DVT: Deep Vein Thrombosis; OR: Odds ratio; 95% CI: 95% Confidence interval.

### Outcome at 30 days post-procedure

The outcomes at 30 days post-operation are shown in Table 3. There were 3 patients in the CAS group who experienced a severe stroke, compared to no patients in the CEA group (2.63% VS 0%, *P* > 0.05). A minor stroke occurred in 6 patients in CAS group and 2 patients in CEA group, respectively (5.26% VS 1.89%, *P* > 0.05). However, the total number of strokes was higher in the CAS group (7.89% VS 1.85%, *P* = 0.038), and OR with 95% CI was 4.54 (1.09–18.97). Although there was no significant difference in mortality in both groups, all of the deaths occurred in the CAS group (1.75% VS 0%, *P* > 0.05); due to a severe stroke after the procedure, one patient died 10 days post-operation, and another patient died 4 weeks post-operation. The incidences of MI after the procedures were similar for the CAS and CEA groups (0.88% VS 0.93%, *P* > 0.05). One patient in each group experienced a MI; both patients had elevated myocardial enzymes but no continuous change in the electrocardiogram. Moreover, the two patients did not show symptoms of angina or MI.

**Table 3:** Outcome at 30 days after procedures.

N (%)	CEA (n = 108)	CAS (n = 114)	*P* value	OR (95% CI)
All stroke	2 (1.85)	9 (7.89)	0.038	4.54 (1.09–18.97)
Severe stroke	0 (0)	3 (2.63)	0.26	N/A
Small stroke	2 (1.89)	6 (5.26)	0.32	N/A
Death	0 (0)	2 (1.75)	0.50	N/A
Myocardial infarction	1 (0.93)	1 (0.88)	0.50	N/A

CEA: Carotid endarterectomy; CAS: Carotid artery stenting; OR: Odds ratio;

95% CI: 95% Confidence interval.

### Outcome of follow-up

All patients were followed up on an outpatient basis, and the recorded data are shown in Table 4. The mean follow-up period was 24.57 ± 13.61 months (range, 3–54 months) in the CAS group and 25.24 ± 12.68 months (range, 3–52 months) in the CEA group. Two patients in the CEA group and three patients in the CAS group were lost to follow-up. During the observation period, 3 patients died after CAS and no patients died after CEA (2.76% VS 0%, *P* > 0.05). Each group had 2 patients who experienced angina pectoris (1.83% VS 1.89%, *P* > 0.05); these patients received further cardiology care.

**Table 4:** The middle term of CEA and CAS procedures.

N (%)	CEA (n = 106)	CAS (n = 109)	*P* value	OR(95% CI)
Total stroke	5 (4.72)	11 (10.09)	0.13	N/A
Ipsilateral stroke	3 (2.83)	9 (8.26)	0.08	N/A
Nonipsilateral stroke	2 (1.90)	2 (1.83)	0.59	N/A
Hemorrhagic stroke	0	0	Null	N/A
Death	0 (0)	3 (2.76)	0.25	N/A
Death/Stroke	5 (4.72)	14 (12.84)	0.036	2.98(1.08–8.23)
Myocardial infarction Restenosis	2 (1.89) 6 (5.66%)	2 (1.83) 15 (13.76)	0.59 0.045	N/A 2.66(1.02–6.74)

CEA: Carotid endarterectomy; CAS: Carotid artery stenting; OR: Odds ratio; 95% CI: 95% Confidence interval.

In the CAS group, three ipsilateral strokes (minor strokes) and two nonipsilateral strokes occurred, with no hemorrhaging or deaths. In the CEA group, nine ipsilateral strokes (8 minor and one disabling) and two nonipsilateral strokes occurred; no hemorrhaging occurred, but there were three deaths. No significant differences were observed for any type of stroke between the two groups. The incidences of total stroke (10.09% VS 4.72%, *P* > 0.05) and ipsilateral stroke (8.26% VS 2.83%, *P* > 0.05) in the CAS group were 2.14 times and 2.92 times the CEA group, respectively, and the incidence of death/stroke were also higher in the CAS group (12.84% VS 4.72, *P* = 0.036), and OR with 95% CI was 2.98 (1.08–8.23).

The long-term outcome was evaluated based on restenosis, which was evaluated after the operation by the same ultrasound team. CAS procedures resulted in a higher restenosis rate (13.76% VS 5.66%, *P* = 0.045), OR was 2.66 with 95% CI (1.02–6.74). The first restenosis without symptoms was detected at 6 months after CAS and at 15 months after CEA. The recurrence of ipsilateral stroke was examined in these restenosis patients, and the relative risk of recurrent stroke in CAS restenosis patients was 1.52 times higher than in CEA restenosis patients; however, this difference was not statistically significant (*P* > 0.05).

## Discussion

The costs of treatment and rehabilitation for stroke and sequelae are enormous economic and social burdens; thus prevention of stroke recurrence is the main purpose of the treatment of carotid artery stenosis^2,3^. Both CEA and CAS are the main methods of treating symptomatic carotid stenosis, although several studies have demnostrated that CAS have the similar clinical effect with CEA procedure, many of these study suffered from a lack of operator experience, patients selection difference. Alternatively, the quality of CAS procedure and baseline data were inconsistent^10,11^. For these reasons, the relative clinical outcomes of CAS and CEA performed by experienced physicians in patients with homogeneous baseline data are unknown. In this study, we clearly defined the surgical experience of the physicians, avoiding potential differences in outcomes. We found that CEA and CAS showed similar mid-term outcomes, whereas CAS had a higher incidence of stroke/TIA at 30 days after the procedure. However, there was no difference in the incidence of MI between the two procedures.

Ischemic stroke caused by carotid stenosis is the local manifestation of cerebral vascular or systemic atherosclerotic disease. In our study, the patients showed a higher incidence of peripheral arterial disease, hyperlipidemia, hypertension, type-2 diabetes and smoking; these high risk factors could affect the outcome of the procedures. Current studies have shown that CEA and CAS are effective in stroke prevention. However, the CEA group showed a higher incidence of MI, and the CAS group showed a higher incidence of stroke. The success rates of both techniques were 100% in the present study, although CAS showed a higher incidence of stroke/TIA at 30 days. However, when the subtypes of stroke were further analyzed, there were no significant differences in the incidences of severe stroke or minor stroke/TIA. This result may be related to the small sample size, although these findings are consistent with the previous reports^12,13^. Stroke after CAS might be related to the stimulation of intraoperative balloon dilatation and CPD movement in the ICA. Moreover, intraoperative plaque loss can lead to fatal or disabling strokes^14^. Stroke after CEA might be related to artery clamping, micro-thromboses in the operative sites or stimulation of the ICA by the carotid shunt. Therefore, the incidence of stroke could be lowered by reducing the use of shunts, increasing the intraoperative blood pressure and enhancing intraoperative monitoring. Although there was no difference in mortality between the CEA and CAS groups, it should be noted that all deaths occurred in the CAS group, which might be associated with a higher incidence of severe stroke after CAS. Moreover, previous reports found that CEA is associated with a higher incidence of MI. The evaluation criteria for these previous studies were primarily elevated myocardial enzymes and an altered ECG; however, coronary artery stenosis was not evaluated. Therefore, some patients with a potentially high risk of MI might have been included in these studies, leading to a biased outcome, which was one of the main reasons why CEA has a higher reported incidence of MI^15^. To avoid potentially biased results, we defined the extent of CAD symptoms and the extent of coronary artery stenosis; thus, MI after CEA and CAS procedures should be directly evaluated. Our study demonstrated that there was no difference in the incidence of MI after the two procedures, a result that differs from those of previous studies.

The most common complication after carotid revascularization is CHS, and a large volume of clinical literature has shown that the incidence of CHS is between 0.44% and 11.7% following CEA and 0.4% to 14% after CAS^16^. In our study, we found that the incidences of CHS were similar after either procedure (11.27% VS 13.89%). All patients showed mild symptoms, such as headache etc. However, after treatment, all of the symptoms disappeared and no cerebral hemorrhage was observed. Because of the stimulation of the carotid sinus during the procedures, hypertension and hypotension were common hemodynamic complications after carotid revascularization. Our results demonstrated that patients showed a higher incidence of hypertension after CEA. However, the patients showed a higher incidence of hypotension after CAS, an effect related to the continuous compression of carotid stents. Post-procedural hypotension is associated with increased post-procedural adverse events. High-risk patients should be aggressively managed to prevent increased morbidity and mortality due to post-procedural hypotension^17^. In addition, the incidence of post-operative hematoma was quite different (from 1.4% to 26%)^18,19^. If treatment is not timely or inappropriate, a secondary surgery is often required, leading to increased medical costs, a longer hospital stay and an elevated rate of complications. Our data showed a similar incidence of hematoma as in previous studies, with no patients developing severe symptoms or requiring secondary procedures. It has been previously reported that CEA might injure the cranial nerve (CN), but a wide range of incidences has been reported (from 2% to 27%). These differences might be associated with the surgical practice, different instruments and the surgical habits of the surgeons^20,21^. In our study, we protected the CN through the use of a standard procedure and microsurgical instruments; only one temporary case of CN palsy occurred, which recovered within 3 months. Furthermore, there were no other complications, such as deep vein thrombosis, incision infection or pseudoaneurysm, although these complications have been previously reported.

Many reports have demonstrated that CEA has better long-term outcomes, but the long-term outcome of CAS has remained controversial. Moreover, a relative higher incidence of stroke and mortality following CAS has been reported^22^. However, a recent study found that the long-term outcome and risk of fatal or disabling stroke were similar for CEA and CAS for the treatment of symptomatic carotid stenosis^23^. In our study, all of the patients were followed up for approximately two years; we found similar mid-term outcomes for the two procedures. Although there were no significant differences in total stroke/TIA, ipsilateral stroke or mortality, CAS showed a higher incidence of total negative events (stroke and death). The long-term outcome of carotid revascularization was affected by the restenosis of the carotid, which can lead to the recurrence of stroke. The SPACE trial showed a restenosis rate of 4.6% in patients undergoing CEA and 10.7% in patients undergoing CAS at 2 years after revascularization, and partial restenosis led to stroke recurrence^24^. Owing to the varying criteria (50% or 70%) of restenosis used in different trials, different restenosis rates after revascularization have been reported. Moreover, the determination of restenosis is also affected by the physician’s experience level and ultrasonic testing^25^.

In this study, we defined restenosis as above 50%, and all of the patients were examined by the same ultrasound team. More restenosis was observed in the CAS group, with the first cases being found 6 months after CAS and 15 months after CEA. Furthermore, although there was no difference between the groups with respect to the recurrence of stroke/TIA, such recurrence was more common in the CAS group. Restenosis within 6 to 24 months was usually attributed to neointimal fibrous hyperplasia, whereas late recurrence after 24 months was chiefly due to the progression of atherosclerosis. However, restenosis after CAS was related to incomplete stent expansion, residual stenosis and radial contraction of the artery^26,27^. In most CAS procedures, we defined residual stenosis less than 30% as a technical success; this definition may have led to an increased restenosis rate given that we defined restenosis as above 50% stenosis. Although self-expanding stents are characterized by a continuous radial expansion force, no data are available regarding whether residual stenosis of the carotid could be alleviated using such a stent, allowing the vessel to return to a normal diameter after the operation^27^. In the present study, we found that restenosis might be associated with the recurrence of a minor stroke/TIA, but a specific relationship between restenosis and recurrent symptoms cannot be determined; therefore, regular follow-ups and ultrasound surveillance are necessary.

In conclusion, we evaluated CEA and CAS using precisely defined selection criteria, experienced surgeons, and homogeneous cases. The results suggested a higher incidence of stroke in the CAS group and no difference in the incidence of MI. CAS showed a higher rate of restenosis. However, due to the limitation of study such as the small sample size, non-randomization and a single center study, the results need to be confirmed with multi-center RCTs.

## Patients and Methods

### Patients

Patients with symptomatic carotid stenosis were retrospective assessed between June 2012 and June 2016. Suitable patients were collected. All of the patients were divided into a CEA or CAS group, and the clinical data were collected and analyzed for a prospective case-control study.

#### Inclusion criteria

(i) The patient was symptomatic for carotid stenosis; (ii) the carotid was severely stenotic (≥70% according to the ECST study^28^); (iii) contralateral stenosis was less than 50%; (iv) there were no symptoms of coronary atherosclerosis disease (CAD); (v) stenosis of the coronary artery was less than 50%; (vi) the carotid anatomy was suitable for CEA and CAS; (vii) the patient was less than 75 years of age; (viii) the vascular doctors had performed more than 200 CEA and CAS surgeries and had surgical experience of at least five years; and (ix) there were no other surgical contraindications.

#### Exclusion criteria

(i) Symptoms of CAD with asymptomatic coronary arterial stenosis of more than 50%; (ii) important organ dysfunction; (iii) uncontrolled hypertension with systolic pressure was more than 180 mmHg; (iv) carotid anatomy was only suitable for CEA or CAS; (v) any hemorrhagic disease within six months; (vi) bilateral carotid stenosis or unilateral carotid occlusion; (vii) intracranial artery stenosis was more than 70%; (viii) fasting blood glucose was more than 11 mmol/L; and (ix) patient refusal.

All of the patients were examined by computational tomography (SOMATOM Definition AS+, Erlangen, Germany) and duplex ultrasound (ATL 3500 HDI; ATL Ultrasound, Bothell, WA, USA). The patients then underwent digital subtraction angiography (DSA), and the cerebrovascular and coronary artery were evaluated. This trial was approved by the local ethics committee and institutional review board; all of the patients provided written informed consent.

### Procedures: CEA procedures

**(**Supplemental Figure I**)** CEA was performed under general anesthesia. When the arotid reflux pressure was below 40 mmHg, the shunt was used. Primary closure or patch closure were performed; a patch was used when the diameter of the intracarotid artery (ICA) was less than 5 mm. The intraoperative monitor procedures was same as CAS procedures. Systemic heparinization (100U/Kg) was used and reversed when carotid anastomosis completed.

### CAS procedures

CAS was operated following a standardized protocol in hybrid operation room equipped with a high-quality imaging system (Axiom Artis FA; Siemens, Erlangen, Germany). On-table rotational angiography was used in selected cases to better visualize the stenosis and to select the optimal working projection. All of the surgeries were performed under local anesthesia. Systemic heparinization (100 U/kg) was administered routinely after the femoral sheath implantation and not reversed at the end of the procedure. Residual stenosis after stenting of less than 30% was defined as a technical success (Supplemental Figure II).

All of the procedures were performed with cerebral protection devices (CPD) and different stent models (open or closed cell, tapered or straight stents). The specific materials used was according to the carotid anatomy and features of lesion. All CPDs were distal filters, namely, Emboshield Filter (Abbott Laboratories, Abbott Park, Ill, USA) (n = 38, 33.3%) or Spide RX Filters (EV3, Plymouth, Minn, USA) (n = 76, 66.7%). Self-expandable stents were of a closed-cell design in 15 cases (13.2%, Carotid Wallstents, Boston Scientific Corp, Natick, Mass, USA); open-cell in 29 cases (Acculink system, Abbott, 25.4%) and 76 cases (Protege Rx Stent System, EV3, 61.4%). Stent size and length were chosen according to preoperative measurements of the target vessel using a Doppler ultrasound scan and by intraoperative measurements using DSA film. Closure devices were used in all CAS cases. In all patients, predilation of the internal carotid lesion was performed with a 3.5–4.0-mm balloon catheter. Post-dilatation using 5-mm balloons was also performed after stent deployment to achieve optimal stent strut position.

### Evaluation and follow-up of patients

All of the patients had been taking aspirin (100 mg) and atorvastatin calcium (20 mg) at least every day since the onset of symptoms or the diagnosis of significant stenosis of the treated ICA; use of this regimen was continued indefinitely. Clopidogrel was started 3 days before the CAS procedure (75 mg daily). Aspirin and clopidogrel were continued for up to 90 days in all CEA and CAS patients, followed by aspirin alone at 100 mg per day. A heparin bolus (5000–10,000 IU) was administered during the procedure. The occurrence of neurologic adverse events during the procedures was recorded. All patients were examined pre- and post-operatively by an independent vascular physician.

The degree and features of stenosis lesion at baseline and during follow-up were evaluated with duplex ultrasound by the same experienced team. “Complex carotid plaque” was determined by ultrasound, the plauqe suggesting irregular plaque surface, uneven echogenicity and a soft appearance of plaque. These results were reconformed by intraoperative evaluation during procedures. The degree of carotid stenosis was determined by standard measurements via angiography.The CT or MR examination was performed for all patients to evaluate whether the new lesions were present.

### Follow-up

Periodic clinical and ultrasound examinations were carried out at outpatient (6, 12 months, and thereafter each year), all symptoms were assessed and recorded.Restenosis (>50%) was deterined by ultrasound team^29,30^. According to the discharge guidance document, patients need to report any new neurological symptoms occurred. Any definite or uncertain neurological symptoms after procedures were assessed by a independent vascular specialist at outpatient. When the revascularization procedures was being reconsidered, the CT angiography or angiography would be performed.

### Clinical Outcomes and Complications

Stroke/TIA were defined as any new neurological event persisting >24 h and included three sub-types: fatal, disabling (mRS ≥ 3) and non-disabling (mRS < 3)^31^. A transient ischemic attack was defined as an acute disturbance of focal neurological function with symptoms lasting less than 24 h and attributed to cerebrovascular disease^32^. Fatal and disabling events were defined as a severe stroke, while non-disabling and TIA were defined as a minor stroke. Myocardial infarction (MI) was defined by the presence of two of the following three criteria: specific cardiac enzymes more than twice the upper limit of normal; a history of chest discomfort for at least 30 min; or the development of specific abnormalities (e.g., Q waves) on a standard 12-lead electrocardiograph^20^. Death or myocardial infarction were defined as procedural if they occurred after stenting or endarterectomy.

The primary outcome was the incidence of any stroke or death at 30 days. The secondary outcomes were any stroke, disabling stroke, TIA, MI, and procedures related complications occurred at 30 days. Late outcomes included the combined endpoint of ipsilateral stroke after procedure; any peri-procedural stroke or death; and the rates of restenosis after procedure.

#### Complications

Surgical complications were defined and recorded as cranial nerve (CN) palsy, hematoma, wound infection, and deep venous thrombosis. Hematoma was defined as documentation of a wound hematoma or wound-related bleeding, as described in previous reports, or the puncture site-related ecchymosis was greater than 5 cm in diameter^18,20^. Cardiac arrhythmias were defined as events requiring antiarrhythmic medication or a pacemaker. Symptomatic hypertension was defined as systolic blood pressure >160 mm Hg or diastolic blood pressure >100 mm Hg lasting >24 h after the operation and requiring medication therapy. Also considered was symptomatic hypotension not associated with bleeding or cardiac failure, with symptoms due to systolic blood pressure of 90 mm Hg requiring administration of a vasopressor agent^33^. Cerebral hyperperfusion syndrome (CHS) was defined as previously reported^34,35^, in brief, the CHS was determined by the detection the blood flow of cerebral artery via TCD and the clinical symptoms of patients, such as headache, dizziness, mental disorders, *et al*. and then excluding new ischemia events and pharmacologic cause.

### Statistical analysis

All of the data were analyzed using SPSS 11.0 (SPSS, Chicago, IL, USA), and *p* < 0.05 was considered statistically significant, and the odds ratio and 95% confidence intervals were calculated to reevaluated the effect of significant difference between CEA and CAS groups. Categorical data were analyzed using a chi-square test, and continuous data were first tested for normality. Normally distributed data are presented as the mean (SD), and the hypothesis significance testing was performed with paired and unpaired t-tests. If the data were not normally distributed, median (interquartile range) values were presented and were analyzed using the Mann-Whitney U test for unrelated samples and the Wilcoxon signed rank test for paired data.

## Electronic supplementary material

Supplemental file
