# Supplementary material for: The middle-term outcome of carotid endarterectomy and stenting for treatment of ischemic stroke in Chinese patients
Source: Sci Rep. 2018 Mar 16;8:4697. doi: 10.1038/s41598-018-23061-7 (PMC5856826; doi:10.1038/s41598-018-23061-7)
Supplement: Supplementary file 1 — Supplemental file [file 41598_2018_23061_MOESM1_ESM.pdf]

The middle-term outcome of carotid endarterectomy and stenting for treatment of ischemic stroke in Chinese patients

LinYang[1],PHD.MD. Jianlin Liu[1], MD. Guangyu Qi[1], Yanzi Li[2], MD. Yamin Liu[1] MD.

1. Department of Vascular Surgery, First Affiliated Hospital of Xi'an Jiaotong University, Xi’an, China
2. Department of Surgery Operation, First Affiliated Hospital of Xi'an Jiaotong University, Xi’an, China

Corresponding: LIN Yang. Department of Vascular Surgery, First Affiliated Hospital of Xi'an Jiaotong University, Xi’an, China. Tel: +86 13572152225. Fax: +86 29 85324050. *E-mail*: jdvascs@163.com

Supplemental Figure I. Process of CEA

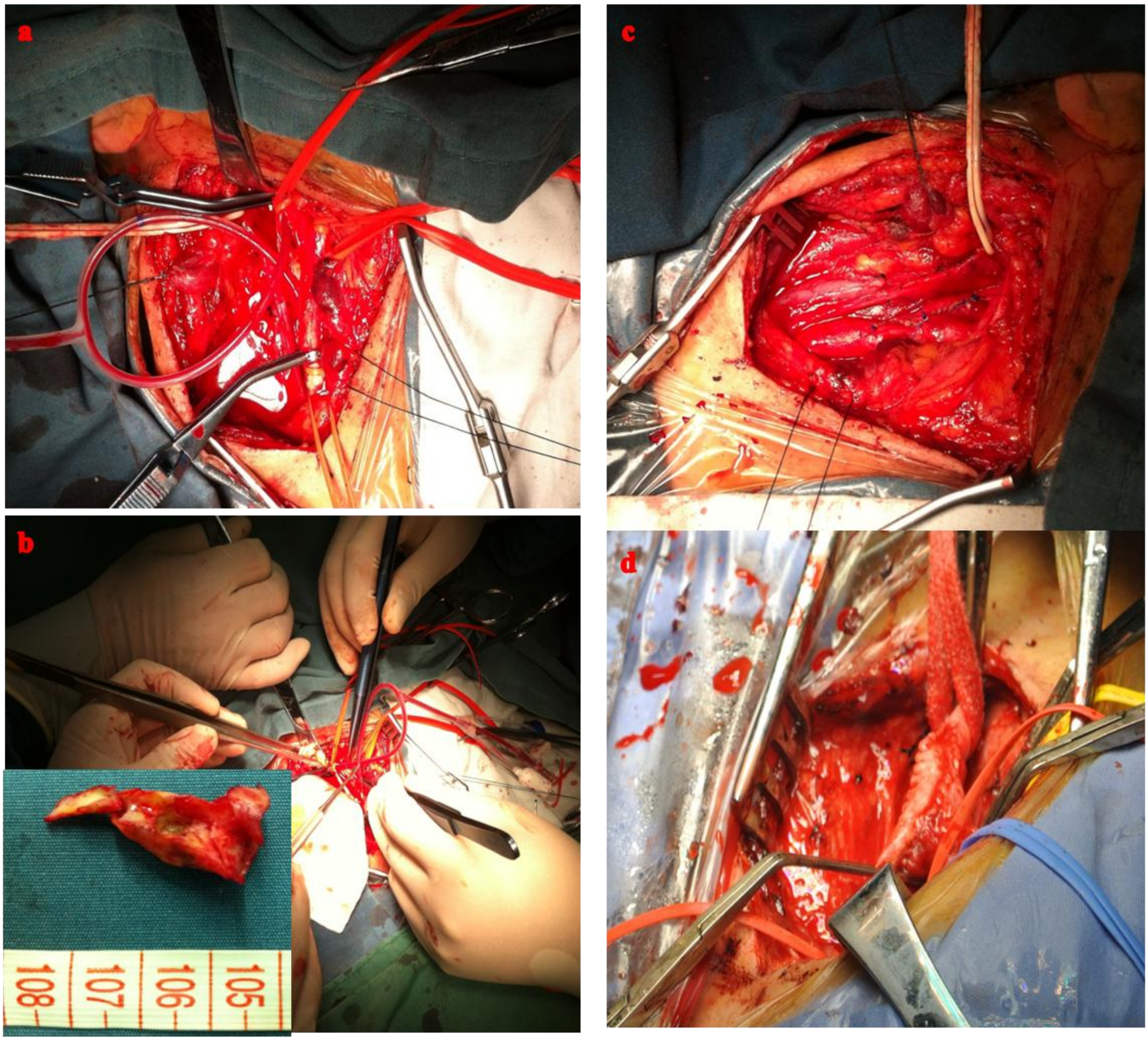

Supplemental Figure II. Process of CAS

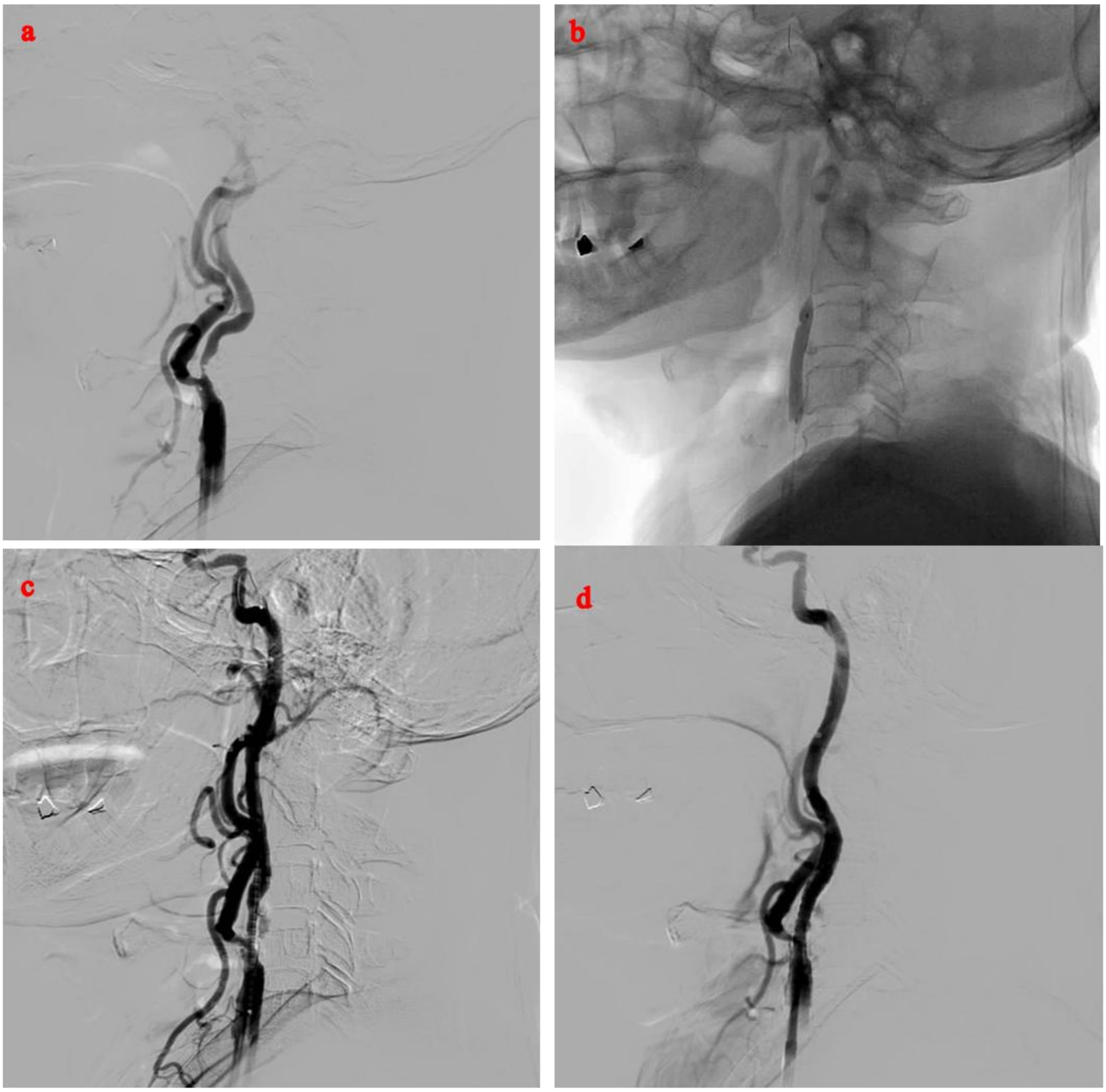

Figure Legends:

Supplemental Figure I. Process of CEA: a) carotid shunting; b) endarterectomy and the Plaque; c) direct anastomosis; d) anastomosis with patch.

Supplemental Figure II. Process of CAS: a) angiography; b) pre-dilation before stenting; c) stenting; d) angiography after stenting.
